# Maize leaf-removal: A new agronomic approach to increase dry matter, flower number and seed-yield of soybean in maize soybean relay intercropping system

**DOI:** 10.1038/s41598-019-49858-8

**Published:** 2019-09-17

**Authors:** Muhammad Ali Raza, Ling Yang Feng, Wopke van der Werf, Nasir Iqbal, Muhammad Hayder Bin Khalid, Yuan Kai Chen, Allah Wasaya, Shoaib Ahmed, Atta Mohi Ud Din, Ahsin Khan, Saeed Ahmed, Feng Yang, Wenyu Yang

**Affiliations:** 1 College of Agronomy, Sichuan Agricultural University, Chengdu, 611130 P.R. China; 2 Centre for Crop Systems Analysis, Wageningen University, PO Box 430, 6700 AK Wageningen, The Netherlands; 3 Maize Research Institute, Sichuan Agricultural University, Chengdu, 611130 P.R. China; 4 College of Agriculture, Bahadur Sub Campus, Bahauddin Zakariya University, Multan, Layyah 31200 Pakistan; 5 College of Life Sciences, Sichuan Agricultural University, Yaan, 625014 P.R. China; 6 College of Food Science, Sichuan Agricultural University, Yaan, 625014 P.R. China

**Keywords:** Light responses, Plant development

## Abstract

Shading conditions adversely affect flower-number and pod-number of soybeans under maize-soybean relay-intercropping (MS_R_). Here we reveal that leaf-removal from maize-canopy improves the photosynthetically active radiation (PAR) transmittance and dry-matter production (DMP) of soybean (especially during the co-growth phase), and compensates the maize seed-yield loss by considerably increasing soybean seed-yield. In a two-year experiment with MS_R_, maize-plants were subjected to different leaf-removal treatments to increase the PAR-transmittance of soybean; removal of the topmost two-leaves (R2), four-leaves (R4), six-leaves (R6), with no-removal of leaves (R0). Leaf-removal treatments improved the PAR-transmittance, photosynthetic-rate, and morphological-characteristics of soybean under MS_R_. At 90 days after sowing, the dry-matter of pods, and seeds was increased by 25%, and 32%, respectively under R6 than R0. Importantly, enhanced PAR-transmittance and DMP under R6 enabled soybean to initiate a greater number of flowers 182.2 plant^−1^ compared to 142.7 plant^−1^ under R0, and it also decreased the flower-abscission (by 13%, from 54.9% under R0 to 47.6% under R6). These positive responses increased the pod-number by 49% and seed-number by 28% under R6 than R0. Overall, under R6, relay-intercropped soybean produced 78% of sole-soybean seed-yield, and relay-intercropped maize produced 81% of sole-maize seed-yield and achieved the land equivalent ratio of 1.59.

## Introduction

**Figure 1:**
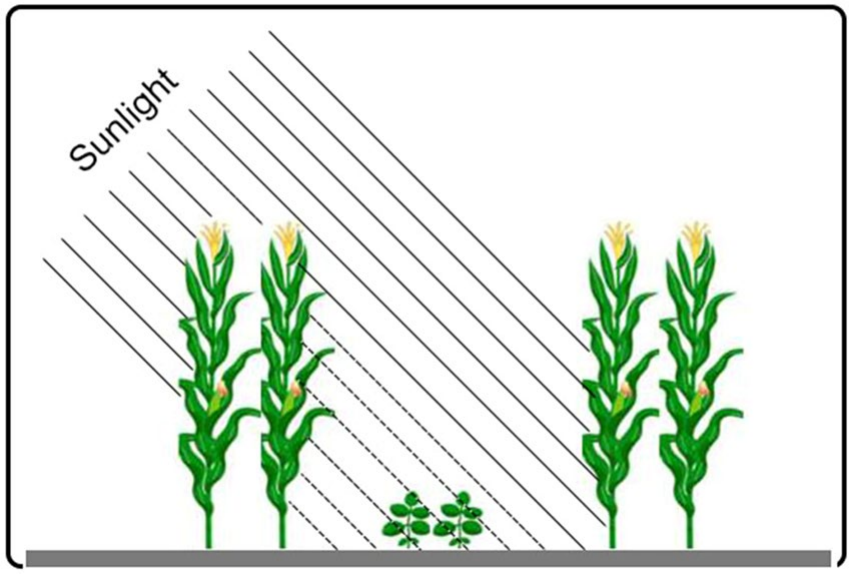
Schematic representation of the maize soybean relay-intercropping system considered in the present experiment.

Maize (*Zea mays* L.) soybean (*Glycine max* L. Merr.) relay-intercropping (MS_R_) is a productive and sustainable cropping system^1,2^. The land equivalent ratio (LER) of MS_R_ is ranged from 1.6 to 1.8^3^, which is higher than that of other relay-intercropping systems in the world^2^. In relay strip intercropping, two crops are planted in the same field in alternating narrow strips of the two species, whereby the growing periods overlap for a limited period of co-growth^4^. Intercropping legumes with cereals are the most common type of relay-intercropping in China, amongst which MS_R_ is most prevalent^5–8^. In MS_R_, soybean is sown 60 days after maize^6^. Therefore, the taller maize plants shade the soybean plants during their early growth (Fig. 1). Soybean is a responsive crop to shading conditions^9^, and under MS_R_ soybean plants suffer from maize shading, especially during their co-growth phase from germination to flowering^8,10^ which increased the seedling height^11^ and reduced the initial dry matter production^12^, stem diameter^13^ and flower initiation of the soybean^14^. Moreover, shaded soybean plants become more susceptible to lodging^15^. The lodging of soybean plants inhibits the translocation of carbohydrates, water, and nutrients, which significantly decreases the soybean seed-yield^16,17^. Reduced growth and flowering of soybean are the main problems of maize soybean relay-intercropping^18^. However, the initiation of flowers and pods under different levels of shading in intercropping was never studied before.

The primary constituents of soybean seed-yield are flower number (flower/plant) and pod number (pod/plant). Genetic improvement or innovation in agronomic practices could enhance future seed-yields by enhancing flower number, pod number, or both constituents. The pod number is directly related to flower number, which shows high environmental variation^19^. The availability of carbohydrates is a major factor controlling flower and pod number^20,21^. Differences in available light for plant growth, dry matter production, and leaf area index have been linked with the variation in final pod number^21–25^. Shading conditions reduce the plant growth rate, decrease the flower, and pod number in soybean^25^. Only a small number of flowers mature into pods with seeds. Shading conditions accelerated the flower abscission, which reduced the final pod number and grain yield^19^. Flower abscission is a major problem of soybean in MS_R_ because it greatly decreases the pod number and grain yield of soybean. It is therefore vital to study how we can decrease the flower abscission in soybean plants during the co-growth phase under MS_R_.

To study the role of shading in the intercropping system on soybean flowering and pod development, we removed maize leaves to increase the light intensity on soybean plants under MS_R_. This study was initiated to investigate the effects of improved light environment on dry matter production and partitioning, flower initiation and abscission, and setting of final pod number in soybean under MS_R_. This critical information of flower initiation and abscission will provide new insights for agronomists to develop the innovative agronomic practices for a higher number of flowers with low abscission rate to prevent the severe yield loss of soybean crop in MS_R_.

## Results

### PAR-transmittance

**Figure 2:**
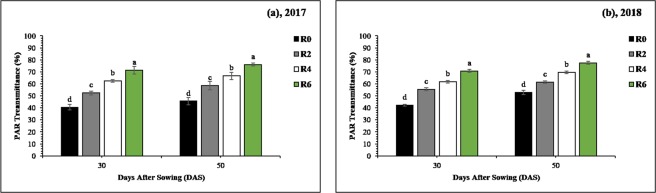
Changes in PAR-transmittance of soybean canopy as affected by leaf removal treatments in 2017 (**a**) and 2018 (**b**). 30 and 70 refer to days after sowing of the soybean crop. The R2, R4, R6, and R0 refer to removal of the topmost two-leaves, four-leaves, six-leaves, with no removal of leaves, respectively, from maize canopy in relay intercropping system. Means are averaged over three replicates. Bars show ± standard errors, (n = 3). Within a bar, different lowercase letters show a significant difference (p ≤ 0.05) between treatments.

Light (photosynthetically active radiation, PAR) transmittance considerably changed under all treatments at soybean canopy in R0, R2, R4, and R6 compared with those of the treatment sole soybean (SS) (Fig. 2). In both study years, the mean PAR-transmittance of soybean canopy in R0, R2, R4, and R6 was always found less than SS at 30 and 50 days after sowing (DAS). However, the leaf removal significantly improved the light environment and the mean maximum PAR-transmittance 71%, and 77% was recorded under treatment R6, whereas the mean minimum PAR-transmittance 41% and 49%, was observed in R0 at 30 and 50 DAS, respectively. After the harvest of maize, non-significant differences were observed for the light intensity between MS_R_ and SS (data not shown).

### Morphological parameters

**Figure 3:**
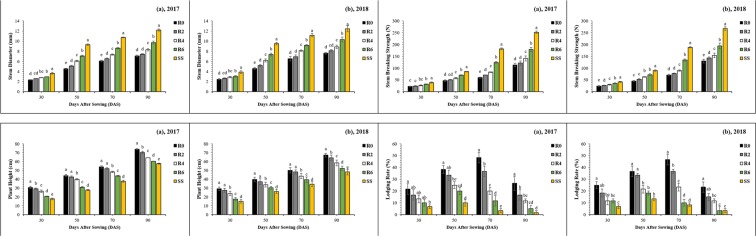
Changes in the stem diameter (**a**,**b**), stem breaking strength (**c**,**d**), plant height (**e**,**f**) and lodging rate (**g**,**h**) of soybean plants as affected by leaf removal treatments in 2017 and 2018. 30, 50, 70 and 90 refer to days after sowing of the soybean crop. The R2, R4, R6, and R0 refer to removal of the topmost two-leaves, four-leaves, six-leaves, with no removal of leaves, respectively, from maize canopy in relay intercropping system. Means are averaged over three replicates. Bars show ± standard errors, (n = 3). Within a bar, different lowercase letters show a significant difference (p ≤ 0.05) between treatments.

The morphological parameters of soybean plants are presented in Fig. 3. Morphology of soybean plants changed significantly under all treatments (P < 0.05 for all the parameters at 30, 50, 70, and 90 DAS). Compared to R0, treatment R6 increased the mean stem diameter (SD) by 19%, 37%, 29% and 27% (Fig. 3a,b), and the mean stem breaking strength (SBS) was enhanced by 32%, 35%, 49% and 35%, (Fig. 3c,d) at 30, 50, 70 and 90 DAS, respectively. At the same sampling times, plant height was decreased by 36%, 27%, 20% and 20% (Fig. 3e,f) in treatment R6 than in R0, and similarly the lodging rate of soybean plants was declined by 54%, 49%, 77% and 83% in R6 as compared with the corresponding values in R0 (Fig. 3g,h). This increase in PAR-transmittance may improve the physiological indices of soybean in MS_R_.

### Leaf area index and photosynthetic characteristics

**Figure 4:**
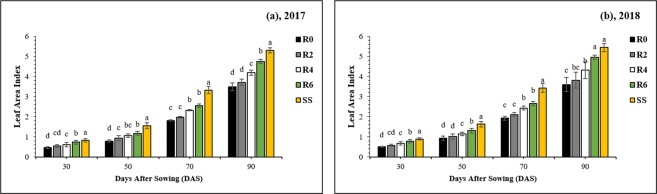
Changes in the leaf area index of the soybean plants as affected by leaf removal treatments in 2017 and 2018. 30, 50, 70 and 90 refer to days after sowing of the soybean crop. The R2, R4, R6, and R0 refer to removal of the topmost two-leaves, four-leaves, six-leaves, with no removal of leaves, respectively, from maize canopy in relay intercropping system. Means are averaged over three replicates. Bars show ± standard errors, (n = 3). Within a bar, different lowercase letters show a significant difference (p ≤ 0.05) between treatments.

Different leaf removal treatments significantly (*P* < 0.05) changed the leaf area index (LAI) of soybean plants from 30 to 90 DAS (Fig. 4). At 90 DAS, relative to SS, the soybean LAI was decreased in R0, R2, R4, and R6 by 34%, 30%, 21%, and 10%, respectively (Fig. 4). Maize leaf removal significantly increased the photosynthesis in soybean (Table 1). It also slightly but significantly increased the transpiration of soybean but lowered the stomatal conductance. The internal CO_2_ concentration was lower in sole soybean and strongly defoliated soybean than in non-defoliated soybean (Table 1). These responses are consistent with the higher amount of light received by sole soybean plants and soybean plants intercropped with strongly defoliated maize plants (R4 and R6), in comparison to non-defoliated (R0) or lightly defoliated maize plants (R2). The effects were consistent between the two years and similar at the two measurement times (30 and 70 DAS).

**Table 1:** Effects leaf removal treatments on photosynthetic characteristics of soybean at 30 (during the co-growth period) and 70 (after the co-growth period) days after sowing (DAS) under sole soybean cropping system (SS) and relay intercropping system in 2017 and 2018.

Years	Treatments	Photosynthetic Rate (μmol CO_2_ m^−2^ s^−1^)		Stomatal Conductance (mol H_2_O m^−2^ s^−1^)		Intercellular CO2 Concentration (μmol CO_2_ m^−2^ s^−1^)		Transpiration Rate (mmol H_2_O m^−2^ s^−1^)	
		30 DAS	70 DAS	30 DAS	70 DAS	30 DAS	70 DAS	30 DAS	70 DAS
2017	R0	13.7e	17.1b	0.60a	0.84a	351.2a	413.3a	2.9d	3.2e
	R2	14.6d	18.0b	0.55ab	0.78ab	331.2ab	381.1ab	3.0d	3.6d
	R4	16.8c	20.6a	0.51b	0.76b	309.6bc	370.7bc	3.2c	3.9c
	R6	18.6b	21.9a	0.44c	0.69c	295.bc	340.1cd	3.3b	4.5b
	SS	19.7a	22.1a	0.42c	0.64c	272.3c	331.3d	3.5a	5.3a
LSD		0.74	1.56	0.05	0.05	39.92	33.08	0.15	0.14
2018	R0	12.8c	17.7c	0.55a	0.72a	332.7a	430.1	3.8e	4.6e
	R2	13.6c	18.6c	0.51ab	0.70a	309.9ab	403.5ab	4.2d	4.9d
	R4	15.7b	20.7b	0.49b	0.66ab	287.7bc	379.7bc	4.3c	5.6c
	R6	17.3b	22.2a	0.42c	0.61bc	270.3cd	352.3c	4.7b	6.2b
	SS	19.2a	22.8a	0.41c	0.58c	246.2d	321.7d	5.3a	7.1a
LSD		1.68	1.12	0.06	0.06	35.04	30.21	0.09	0.13

The R2, R4, R6, and R0 refer to removal of the topmost two-leaves, four-leaves, six-leaves, with no removal of leaves, respectively, from maize canopy in relay intercropping system. Means are averaged over three replicates. Means do not share the same letters in the column differ significantly at p ≤ 0.05.

### Dry matter accumulation

**Figure 5:**
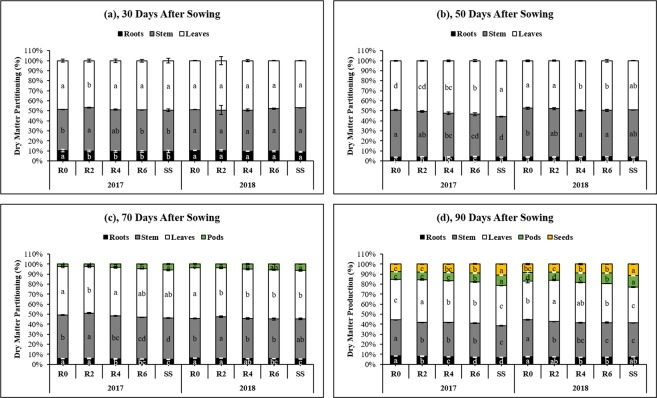
Dry matter partitioning to root, stem, leaves, pods, and seeds of soybean plants as affected by leaf removal treatments in 2017 and 2018. 30, 50, 70 and 90 refer to days after sowing of the soybean crop. The R2, R4, R6, and R0 refer to removal of the topmost two-leaves, four-leaves, six-leaves, with no removal of leaves, respectively, from maize canopy in relay intercropping system. Means are averaged over three replicates. Bars show ± standard errors, (n = 3). Within a bar, different lowercase letters show a significant difference (p ≤ 0.05) between treatments.

Maize leaf removal had a significant impact on dry matter accumulation of soybean (Table 2). Soybean plants in sole cropping system obtained higher dry matter than those in R0, R2, R4, and R6 under MS_R_. Amongst the leaf removal treatments, R6 produced the highest dry matter (60.1 g plant^−1^ at 90 DAS in 2017 and 68.3 g plant^−1^ in 2018) of soybean. The soybean total dry matter demonstrated the trend SS > R6 > R4 > R2 > R0, indicating that increased the PAR-transmittance at soybean canopy in relay intercropping system considerably enhanced the soybean dry matter accumulation by reducing the adverse effects of maize shading on soybean growth. Moreover, the different leaf removal treatments significantly (*P* < 0.05) altered the dry matter distribution among root, stem, leaves, pods, and the seed of soybean (Fig. 5). At 30 and 50 DAS, the maximum proportion of dry matter distribution was noticed in leaves followed by stem and root in R0, R2, R4, R6 and SS (Fig. 5a,b). However, at 70 and 90 DAS, the pattern of dry matter partitioning was changed, and soybean plants invested their dry matter in pod and seed formation. At 90 DAS, relative to R0, the proportion of pods and seeds dry matter increased by 13%, 46%, and 87%, and 14%, 45%, and 72%, respectively in R2, R4, and R6 (Fig. 5c,d).

**Table 2:** Effects leaf removal treatments on dry matter production, flower initiated and flower abscission of soybean at 30, 50, 70 and 90 days after sowing (DAS) under sole soybean cropping system (SS) and relay intercropping system in 2017 and 2018.

Years	Treatments	Dry Matter Production (g plant^−1^)				Flower Initiated (plant^−1^)	Flower Abscission (% plant^−1^)
		30 DAS	50 DAS	70 DAS	90 DAS	50–90 DAS	50–90 DAS
2017	R0	3.6e	17.3e	25.0e	38.0e	133.4d	54.5a
	R2	4.2d	18.5d	29.2d	44.4d	146.9cd	53.2ab
	R4	4.7c	20.9c	34.7c	51.7c	165.8bc	52.3abc
	R6	5.2b	22.8b	38.8b	60.1b	179.1b	48.4bc
	SS	5.9a	26.8a	45.3a	70.1a	207.3a	46.7c
LSD		0.09	0.99	1.07	1.55	21.61	6.07
2018	R0	4.0c	18.5e	28.8e	44.1e	152.0c	55.2a
	R2	4.4c	20.7d	33.0d	51.d	160.3c	52.6ab
	R4	5.0b	22.6c	37.7c	58.6c	176.4b	51.1ab
	R6	5.5b	25.5b	41.7b	68.3b	185.4b	46.8b
	SS	6.5a	31.3a	48.1a	76.0a	218.3a	45.7b
LSD		0.52	1.02	1.10	2.93	14.12	7.18

The R2, R4, R6, and R0 refer to removal of the topmost two-leaves, four-leaves, six-leaves, with no removal of leaves, respectively, from maize canopy in relay intercropping system. Means are averaged over three replicates. Means do not share the same letters in the column differ significantly at p ≤ 0.05.

### Flower initiation and abscission

**Figure 6:**
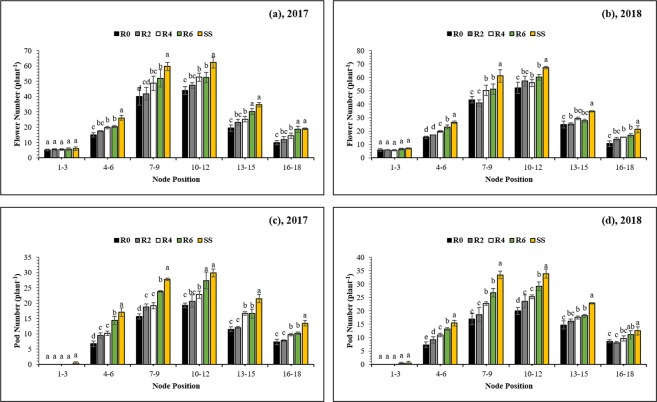
Flower number (**a**,**b**) and pod number (**c**,**d**) of each soybean node as affected by leaf removal treatments in 2017 and 2018. The R2, R4, R6, and R0 refer to removal of the topmost two-leaves, four-leaves, six-leaves, with no removal of leaves, respectively, from maize canopy in relay intercropping system. Means are averaged over three replicates. Bars show ± standard errors, (n = 3). Within a bar, different lowercase letters show a significant difference (p ≤ 0.05) between treatments.

In MS_R_, soybean plants were co-planted with maize plants in the field from germination to flowering. During this period, soybean plants suffered from maize shading, flower initiation and abscission significantly affected as compared to the sole cropping system. Flowering was initiated earlier in intercropping without any defoliation (R0) (51 DAS in 2017 and 54 DAS in 2018) (Table 3) than in sole soybean (56 DAS and 59 DAS in 2017 and 2018, respectively). Likewise, flowering was terminated earlier in intercropping without any defoliation (R0) (94 DAS in 2017 and 96 DAS in 2018) (Table 3) than in sole soybean (99 DAS and 101 DAS in 2017 and 2018, respectively). Defoliation of maize leaves resulted in a later onset and termination of flowering in soybean than in the intercropping without defoliation, demonstrating that shading by maize advances and shortens the flowering period in soybean. In SS, the average number of flowers initiated (49%) and abscised (16%) were significantly higher and lower, respectively as compared to R0 treatment in both years (Table 2). Maize leaf removal significantly increased the number of flowers initiated, and it reduced the flower abscission rate in MS_R_. Overall, soybean plants in R6 produced a higher number of flowers than R0 (by 34% in 2017 and 22% in 2018) and reduced the flower abscission (by 13% in 2017 and 18% in 2018) (Table 2). Thus, maize leaves defoliation increased flower production in soybean and reduced the flower abscission rate, resulting in a higher number of pods. In addition, we also measured the total number of flowers initiated at each node. The initiated flowers were significantly (*P* < 0.05) higher at middle nodes (9^th^ to 12^th^ node of the main stem) than at upper and lower nodes (Fig. 6a,b). The initiated flowers per node of relay-intercropped soybean were significantly (*P* < 0.05) lower than that of sole cropping soybean. Flowers initiation of treatment R6 was significantly (*P* < 0.05) higher than that of R0 at the 7th–12th node of the main stem (Fig. 6a,b).

**Table 3:** Effects leaf removal treatments on total pod number, seed number, 100-seed weight, seed-yield, and land equivalent ratio (LER) of maize and soybean under sole soybean cropping system (SS), sole maize cropping system (SM) and relay intercropping system in 2017 and 2018.

Years	Treatments	Pod Number	Seed Number	Seed Weight	Seed-Yield (kg ha^−1^)		LER		Total LER
		(plant^−1^)	(plant^−1^)	(g)	Soybean	Maize	Soybean	Maize	
2017	R0	60.4e	79.3d	17.73^NS^	1406.1d	6834.5b	0.56c	0.89b	1.45b
	R2	68.6d	85.4cd	17.72	1512.7cd	8353.2a	0.60bc	1.10a	1.69a
	R4	78.5c	92.4c	17.71	1633.2c	6513.1b	0.65b	0.85b	1.50b
	R6	92.3b	103.4b	17.70	1831.3b	6167.8b	0.73a	0.80b	1.53b
	SS	110.1a	142.4a	17.68	2523.3a	—	—	—	—
	SM					7713.9a	—	—	—
LSD		5.65	7.29	0.23	138.08	819.81	0.06	0.11	0.10
2018	R0	67.5e	94.4d	17.51^NS^	1650.4d	6634.9c	0.66c	0.92b	1.58c
	R2	75.5d	100.7cd	17.49	1761.0cd	7666.1a	0.70bc	1.06a	1.77a
	R4	86.2c	107.1c	17.48	1867.5c	6228.2d	0.75b	0.86c	1.61bc
	R6	98.8b	119.5b	17.46	2091.6b	5904.7e	0.84a	0.82d	1.65b
	SS	118.6a	142.8a	17.45	2496.5a	—	—	—	—
	SM					7238.3b	—	—	—
LSD		5.81	6.90	0.53	153.66	264.64	0.05	0.05	0.06

The R2, R4, R6, and R0 refer to removal of the topmost two-leaves, four-leaves, six-leaves, with no removal of leaves, respectively, from maize canopy in relay intercropping system. Means are averaged over three replicates. Means do not share the same letters in the column differ significantly at p ≤ 0.05.

### Yield and yield components

In both years, different leaf removal treatments significantly (*P* < 0.05) affected the soybean seed-yield (Table 3). On average over the two years, relative yield per plant compared to sole soybean was 0.61 in R0, 0.65 in R2, 0.70 in R4 and 0.78 in R6. Among the intercropping treatments, R6 had a maximum average soybean grain yield (1961.5 kg ha^−1^) while R0 had minimum average soybean grain yield (1528.2 kg ha^−1^). On average, as compared to R0, treatments R2, R4, and R6 increased the grain yield of soybean by 7%, 15%, and 28% in both years. In addition, relative yield per plant compared to sole maize was 0.90 in R0, 107 in R2, 0.85 in R4 and 0.81 in R6. (Table 3). On average, treatments R4 and R6 decreased the seed-yields of maize by 6% and 12%, while R2 enhanced the maize grain yield by 19% than R0 in both years. Average across the two cropping seasons, SS significantly produced the highest pod number per plant (114.4 plant^−1^) and the highest grain number of a plant (142.6 plant^−1^). There were non-significant differences between treatments in the individual seed weight (mg) of soybean. However, different levels of leaf removal exhibited a significant impact on the final pod number plant^−1^ and the seed number plant^−1^, with highest values of 95.6 pods and 111.5 seeds plant^−1^ recorded under R6, followed by R4 (82.4 pods plant^−1^ and 99.8 seeds plant^−1^) and R2 (72.1 pods plant^−1^ and 93.0 seeds plant^−1^), and lowest values (63.9 pods plant^−1^ and 86.9 seeds plant^−1^) in R0. Pod number was higher at the middle nodes (8th-13th nodes of the main stem) than at the upper and lower nodes (Fig. 6c,d). The pod number per plant of SS was significantly higher than that of relay-cropped soybean at each node of the main stem.

### Land equivalent ratio

In this study, the land equivalent ratio values of maize (LERm) were found higher than the corresponding land equivalent ratio values of soybean (LERs) in all leaf removal treatments. However, the land equivalent ratio (LER) of soybean was considerably improved in R2, R4, and R6 as compared to R0 treatment. In addition, treatments R2, R4, and R6 increased the LER of soybean by 7%, 17%, and 31% in 2017 and 6%, 13%, and 27% in 2018 as compared to R0, suggesting that LERs are closely related to the changes of light environment. Overall, under MS, the mean LER value of treatment R2 (1.73) was significantly higher than R0 (1.51), R4 (1.55) and R6 (1.59) in both years (Table 3).

## Discussion

### Effects of improved light environment on morphological characteristics

The light intensity of soybean plants decreases in MS_R_ as compared to sole cropping of soybean (SS)^18^. However, in the current experiment, different levels of leaf removal in MS_R_ enhanced (from 41% to 77%) the PAR-transmittance of soybean canopy by decreasing the rate of light absorbed and reflected by maize leaves which in turn improve the PAR-transmittance of soybean plants in MS_R_ (Fig. 2). Previously, many studies have revealed that the lodging rate and plant height of soybean plants increases under shading conditions^26,27^. However, in this experiment, improved PAR-transmittance of soybean canopies led to decrease in the lodging rate and plant height of soybean plants under MS_R_ (Fig. 3). Moreover, the SD and SBS of soybean are of primary concern for agronomist because these indices were adversely affected in intercropping conditions^28^. Similarly, our results confirmed that the decrease in SD and SBS of soybean plants under MS_R_ is the consequence of dense maize shade during the co-growth phase. Therefore, increased SD and SBS of soybean plants are more favorable to reduce the soybean lodging in MS_R_.

### Effects of improved light environment on leaf area index and photosynthetic characteristics

Improvement in leaf area index can improve the crop performance by enhancing the photosynthetic-rate^29^. At all sampling times, the leaf area index of soybean achieved the highest value in R6, followed by R4, R2, and R0 (Fig. 4). Higher LAI of soybean resulted from improved light environment exhibits the optimum leaf expansion and development, which increased the light-harvesting and efficiency of soybean leaves. This improvement in leaf area index might be due to the improved light environment of soybean canopy, which significantly improved the LAI of soybean because soybean LAI under MS_R_ is directly related to the available light intensity^30,31^. The photosynthetic-rate of soybean leaves changed significantly under different light levels^13^, and shading conditions decreased the soybean photosynthetic-rate under MS_R_^4^. In this experiment, increased PAR-transmittance of soybean canopy from R2 to R6 in MS_R_ led to enhance the transpiration- and photosynthetic-rate of soybean plants, but reductions were measured for intercellular CO_2_ concentration and stomatal conductance of soybean leaves in MS_R_ (Table 1). These results are indicating that the adequate light can improve the photosynthetic characteristics of soybean leaves by decreasing the adverse impacts of maize-shading in MS_R_. These findings are consistent with the previous studies in which researchers have revealed that crop plants alter their physiological characteristics to perform better under the prevailing conditions^32–34^.

### Effects of improved light environment on dry matter accumulation

The increased leaf area and photosynthetic-rate are the main factors for dry matter production (DMP) in oilseed crops^35^. Previously, researchers had confirmed that the decreased PAR-transmittance significantly reduced the DMP of soybean plants under MS_R_^12^. However, our results demonstrated that the leaf removal from maize canopy during the co-growth period in MS_R_, soybean adequately harvested and used enough sunlight for their biochemical and physiological processes which maintained the higher DMP. The treatment R6 significantly increased mean DMP of soybean by 40%, 35%, 50%, and 56% at 30, 50, 70, and 90 DAS, respectively than R0 (Table 2). The higher DMP in soybean might be due to the adequate accumulation of major nutrients from germination to maturity in MS_R_ because an enhanced accumulation of major nutrient directly increases the intercrop DMP^36,37^ and improve light intensity significantly enhanced the nutrient uptake in crops^38^. Furthermore, the dry matter partitioning changed considerably in soybean, and our findings of treatment R0 confirms the past results which conclude that shading conditions significantly increase the dry matter allocation to stem, decreases the distribution of dry matter to roots and leaves in soybean^12,39^. However, the improved PAR-transmittance under R4 and R6 balanced the dry matter distribution between vegetative parts (stem and leaves) and reproductive parts (pods and seeds) in MS_R_ (Fig. 5).

### Effects of improved light on flower initiation and abscission

The main flush of flower initiation was directly affected by PAR-transmittance of soybean canopy in all leaf removal treatments. The initiated flower number accelerated proportionately with the increase in PAR-transmittance (Fig. 2) and leaf area index (Fig. 4) in R0, R2, R4, and R6 treatments for both years. Remarkably, the flower initiation was sustained at a high rate under treatment R6 where soybean plants accumulated higher dry matter than other treatments irrespective of full sunlight availability in all treatments after the harvest of maize crop in MS_R_, which suggested that flower initiation in soybean plants is sensitive to initial growth and dry matter accumulation. Moreover, the total number of initiated flowers increased concomitantly in soybean plants with the enhanced leaf growth. Indeed, a higher number of flowers would require increased partition of current photo-assimilates to flowering buds. As both, leaf growth and flower initiation increased with the increase in PAR-transmittance in MS_R_; it was evident that competition for photo-assimilates did not impinge on flower initiation. Previously, scientists have reported that the developing reproductive parts need a large amount of photo-assimilates to maintain growth during their initial developmental stages^40,41^. Therefore, photo-assimilate limitation seems to impinge at this early stage. Additionally, treatment R6 in MS_R_ significantly reduced the flower abscission rate (Table 2), and this reduction in flower abscission may be due to the utilization of stored assimilates for flower initiation^14^. Our results are consistent with previous findings in which researchers had reported that the flower abscission was decreased when soybean plants were opened artificially at flowering stage^42^, while higher flower abscission in soybean was recorded under shading conditions^19^.

### Effects of improved light on yield and yield components, and land equivalent ratio

The final pod-number at harvest was the outcome of the balance between two components: total initiated flowers and total abscised flowers. In this study, leaf removal treatments significantly increase the final pod-number of soybean plants at soybean harvest through increase the flower initiation and decrease the flower abscission. This response was predominantly due to the increase PAR-transmittance impact on flower initiation, which was positively linked with the assimilatory capacity of soybean plants. Similarly, except under R0, all leaf removal treatments significantly enhanced the final pod-number through the decrease of flower abscission because, in this way, most of the flower developed into pods. Previous predictive models of final pod-number^43–45^, and recent model which incorporates the flowering temporal profile^46^ are carbohydrate-based models. Present findings regarding the flower abscission component of the final pod-number per plant could be described by carbohydrate-based models. The seed-number (plant^−1^) showed the significant differences among different treatments and was affected by the initial growth and DMP, which provide the photo-assimilate for pod and seed formation. This finding suggested that seed-number in soybean plants was under the control of total leaf area and carbohydrate production^47^ and our leaf removal treatments significantly enhanced the source size which may be the possible reason of higher seed number in R6 under MS_R_ (Table 3).

Furthermore, we observed the non-significant differences for seed weight of soybean in all treatments (Table 3) which might be due to the genetic character of soybean variety (Nandou-12), consistent with a past report where scientist concluded that seed size is genetically determined^48^. We further investigated the impacts of leaf removal levels on soybean seed-yield under MS_R_ and SS. Our findings showed that the mean maximum soybean seed-yield was produced under treatment SS, while in leaf removal treatments under MS_R_, the average highest soybean seed-yield was measured in R6, with an improvement of 30% in 2017 and 27% in 2018 as compared to R0 (Table 3). This improvement in the seed-yield of soybean might be attributed to the higher pod and seed number, which all together enhanced the soybean seed-yield in MS_R_. On average, mean soybean seed-yield was increased by 28%, and maize seed-yield was decreased by 12% in R6 as compared to their corresponding values in R0. On the other hand, treatment R2 was the only treatment which increased the seed-yields of both intercrop species in MS_R_. This improvement in seed-yields of intercrop species maybe because of the optimum growing conditions^8,49^, better light interception within maize canopy^50^ and at soybean canopy, delayed leaf senescence at maturity^51,52^, and adequate uptake of major nutrients^8,53^. Moreover, the improved light conditions increased the nitrogen fixation ability of soybean plants^54^ and leaf defoliation treatments considerably improved the PAR-transmittance at soybean canopy. Therefore, it is possible that in MS_R_, the neighboring deep-root maize plants accumulated extra nitrogen that was released by soybean nodule^3^, which enhanced the maize seed-yield under R_2_ in both years. In addition, non-significant differences were noticed for the maize seed-yield under treatments R4, R6, and R0 in 2017. However, as compared to R0, R4 and R6 decreased the maize seed-yield by 6% and 12% in both years. This reduction in maize seed-yield under R4 and R6 might be due to the reduced photosynthesis at maturity due to the severe leaf loss (Data not shown)^51^ and decreased translocation of dry matter towards maize cob^55^, similar results were reported in past studies^6,56^.

Additionally, in this experiment, the values of LER were always greater than one under all treatments in MS_R_, which shows the seed-yield advantage of MS_R_ over sole soybean and maize cropping systems due to the improved exploitation of water, land, light, and nutrients for growth^57^. Specifically, the mean values of LER in R0, R2, R4 and R6 were 1.51, 1.73, 1.55 and 1.59, respectively (Table 3), which shows that 51% to 73% of more agricultural land will be required by sole crop of soybean and maize to equal the seed-yield of MS, demonstrating the greater land-use efficiency than SS and SM^58^. Overall, the removal of leaves from maize top under MS_R_ significantly improves the initial growth and development of soybean plants, accelerated the DMP and flower initiation in soybean and compensated the maize seed-yield loss by substantially increasing the seed-yield of soybean plants in MS_R_.

## Conclusion

In the present study, our results revealed that different leaf defoliation levels had positive effects on soybean growth and development in MS_R_. The PAR-transmittance of soybean canopy was strengthened, and morphological characteristics of soybean were improved significantly for R4 and R6 treatments, which increased the LAI and photosynthetic rate of soybean plants, and soybean obtained a greater seed-yield under MS_R_. The most important finding of this study was the demonstration that the number of initiated flowers increased and the rate of flower abscission decreased as the PAR-transmittance, and dry matter production of soybean plants increased in MS_R_, especially during the co-growth period. This response exhibited a similar trend in all leaf removal treatments despite significant differences between SS and MS_R_. Moreover, heavy defoliation (R4 and R6) decreased the maize seed-yield and considerably increased the soybean seed-yield in MS_R_. Interestingly, treatment R2 was the only treatment which increased the seed-yield of both intercrops in MS_R_. Further experiments are needed to understand the nature of the internal signals modulating flower initiation.

## Methods

### Research location and planting material

This study was conducted in 2017 and 2018 at the research area of Sichuan Agricultural University in Ya’an (29° 59′ N, 103° 00′ E, altitude 620 m), China. We used the semi-compact Zhenghong-505 maize variety and the shade-tolerant Nandou-12 soybean variety in both years of experimentations. Both varieties are widely used for the production of soybean and maize in southwestern parts of China^11^.

### Weather conditions and soil properties

The experimental site has a subtropical and humid climate. Long-term (1960–2010) average temperature is 16.21 °C, and average precipitation is 1200 mm. The weather data of experimental location during the growing seasons from 2017 to 2018 is presented in Table 4. The soil characteristics at Yaan were: pH of 6.62, organic matter of 29.85 g kg^−1^, total N of 1.62 g kg^−1^, total P of 1.28 g kg^−1^, total K of 16.32 g kg^−1^, available N of 317.14 mg kg^−1^, available P of 42.26 mg kg^−1^, and available K of 382.14 mg kg^−1^ in 0–30 cm soil layer.

**Table 4:** Monthly rainfall, average temperature, and humidity from March to October in the growing seasons of 2017 and 2018.

Month	Years					
	2017			2018		
	Rainfall (mm)	Average T (°C)	Humidity (%)	Rainfall (mm)	Average T (°C)	Humidity (%)
March	41.1	15.63	56.34	26.7	14.11	55.37
April	65.5	19.39	62.27	53.5	19.14	57.31
May	93.7	22.45	66.31	113.1	23.52	56.36
June	167.1	26.41	61.37	151.7	25.54	56.45
July	205.7	27.73	84.43	185.4	29.19	62.39
August	126.8	28.66	65.99	223.6	27.72	80.14
September	172.5	22.32	79.21	146.3	23.55	54.35
October	21.4	19.48	57.29	59.4	17.68	77.87
March-October	893.8	22.75	66.65	959.7	22.56	62.53

### Experimental design and details

**Figure 7:**
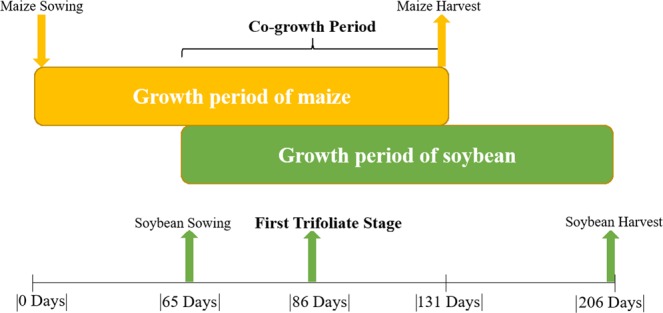
Illustration of the growth period of maize soybean relay-intercropping system. The upper-bar shows the growing period of maize crop (131days, first sown relay-crop), and the lower-bar shows the growing period of soybean crop (141 days, second sown relay-crop). The co-growth period (66 days), is defined as the proportion of the total system growth period that both relay-crops grow together.

A randomized complete block design (RCBD) with four-leaf removal treatments and three replicates was used in both years of experiments. Soybean was relay intercropped with hybrid maize, and the results were compared with sole soybean (SS) and sole maize (SM). The MS_R_ was used in the present study. In this experiment, we used the same planting arrangements which farmer practices in this area, and each strip in MS_R_ contained 2 maize rows and 2 soybean rows (2:2). In this system, row to row spacing between soybean to soybean and maize to maize rows was 40 centimeters, and the gap of 60 centimeters was maintained between the rows of soybean and maize. Furthermore, sole soybean and maize were sown with a row distance of 50 centimeters and 70 centimeters, respectively. The size of each plot was 6 meters wide by 6 meters long. Both varieties were overseeded and thinned to keep a plant population of 100000 and 60000 plants per hectares for soybean and maize, respectively. Similar plant population was maintained in SS and SM by keeping a distance of 20 centimeters and 16.7 centimeters for soybean and maize, respectively. The times of sowing were the first week of April for maize and the second week of June for soybean in both years. The times of harvesting were the second week of August for maize and the last week of October for soybean in both years, and the cogrowth phase is shown in (Fig. 7). Basal fertilizers were applied at the time of sowing in intercropped and sole-cropped maize at a rate of N, P, and K of 135, 72, and 90 kg ha^−1^, respectively. The second dose of N for maize was applied at 75 kg ha^−1^ in all plots. Soybean was fertilized at sowing at rates of N, P, and K of 75, 40, and 4 kg ha^−1^, respectively. For fertilizers, urea, calcium superphosphate, and potassium chloride were used as sources of N, P, and K, respectively. All other farming practices were kept the same in all plots according to the crop demand and farmer’s practices in the area^4^. The crops were rainfed.

### Treatments

**Figure 8:**
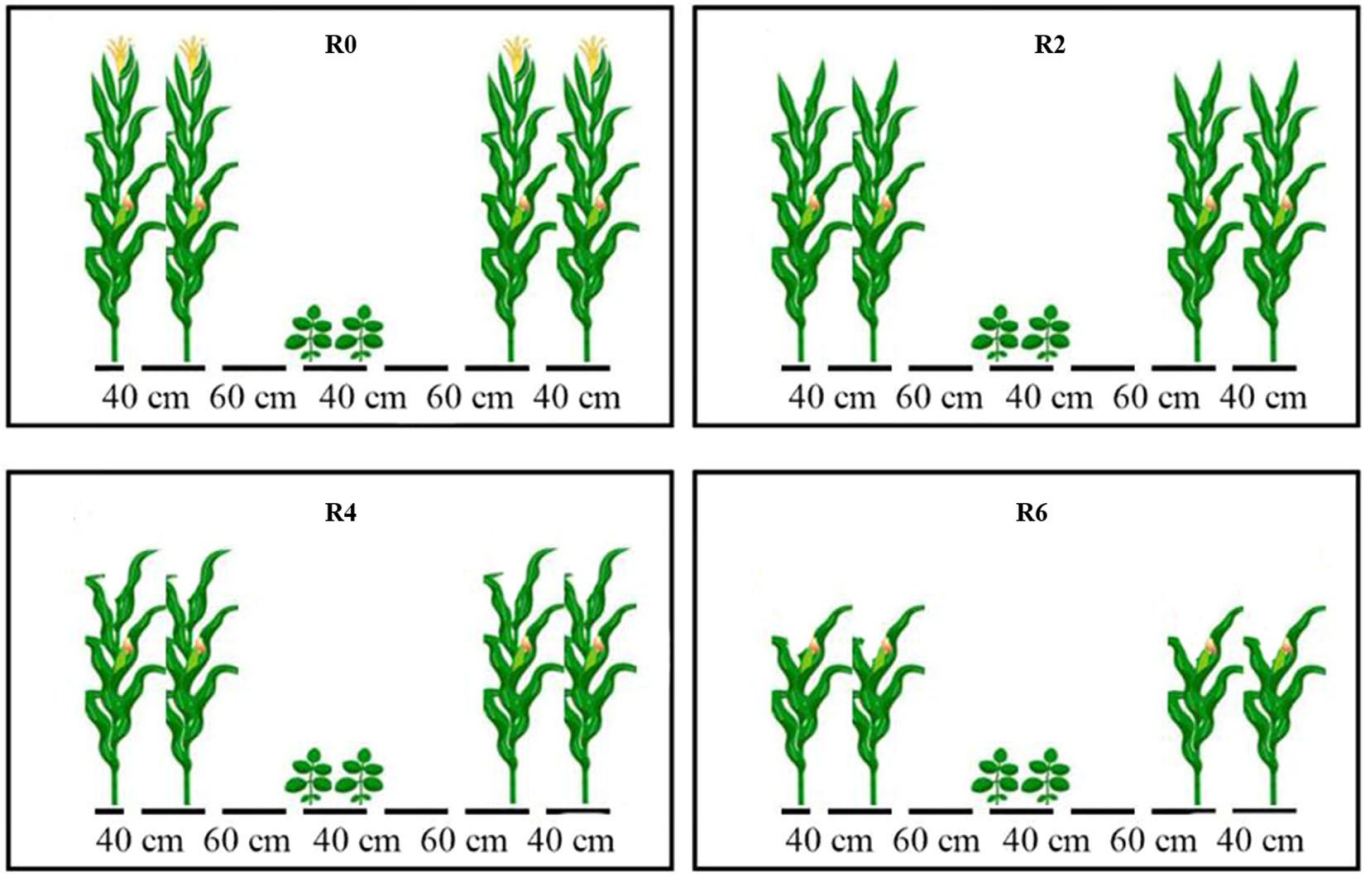
Schematic representation of maize canopy as affected by leaf removal treatments in 2017 and 2018. The R2, R4, R6, and R0 refer to removal of the topmost two-leaves, four-leaves, six-leaves, with no removal of leaves, respectively, from maize canopy in relay intercropping system.

Leaf removal treatments were applied to maize, when the maize was at silking stage (30^th^ June 2017 and 28^th^ June 2018), and soybean at the first trifoliate leaf stage (Fig. 8) viz. no removal of leaves (R0); removal of two-leaves (R2); removal of four-leaves (R4); removal of six-leaves (R6) from the canopy of maize plants in MS_R_.

### Measurements

#### Light conditions

Photosynthetically active radiation (PAR) was measured in all treatments to determine the variations of PAR-transmittance at soybean canopy. LI-191SA quantum sensors were placed on the horizontal arm of an observation scaffold, first above of maize canopy, and subsequently of soybean canopy. This was done at 30 and 50 days after sowing (DAS) in all treatments and all replicates during the co-growth period. In each treatment, the PAR was recorded three times on soybean and maize top from 10:00 am to 1:00 pm on a clear day, and then the average was measured. From the data, we calculated PAR-transmittance to the soybean as a percentage of incoming light at the top of the maize plants.

#### Morphological parameters

At 30, 50, 70 and 90 DAS, twenty soybean seedlings from each treatment were chosen, then plant height was determined from ground to top, and stem diameter was measured by using a Vernier caliper. Soybean’s lodging rate was assessed by determining the proportion of soybean stems (20 soybean stems per plot) for which the angle between the soybean stem and the ground was less than 30 degree^11^. The digital plant lodging tester was used to measure the stem breaking strength of soybean plants^18^.

#### Leaf area index and photosynthetic characteristics

The leaf area index of soybean in different treatments was determined at 30, 50, 70, and 90 DAS. For this purpose, ten consecutive soybean plants were sampled destructively from each treatment. The maximum leaf length and width were determined using a ruler. Then the leaf area was calculated by multiplying the leaf length, leaf width, and crop-specific coefficient factor of 0.75 for soybean^59^. Photosynthetic characteristics of soybean leaves were determined using a Li-6400 portable photosynthesis system equipped with a LED leaf chamber^35^. In all treatments, 3 expanded leaves from each experimental plot from soybean canopy were selected at 50 and 70 DAS, and photosynthetic characteristics were measured from 10:00 to 11:00 am on a clear day under a CO_2_ concentration of 400 µmol mol^−1^.

#### Dry matter accumulation

To measure the total dry matter accumulation and distribution over different plant parts (g plant^−1^), ten consecutive soybean plants were harvested from all treatments at 30, 50, 70 and 90 DAS. All samples were divided into root, stem, leaves, pods, and seed. All subsamples were oven-dried at 105 °C for one hour to kill the fresh-tissues and then dried at 65 °C until a constant weight reached. Cores (15 centimeters height and 8 centimeters diameter) for root dry matter analysis of soybean were collected using a core sampler. The soil cores were immersed in tap water and kept for three hours to disperse the soil. A root washer then separated roots from the dispersed soil.

#### Flower initiation and abscission

Flower formation and flower abscission were monitored on a daily basis from 51 DAS to 94 DAS on ten plants in each plot and replication. All nodes on all stems on a plant were carefully inspected, and new open flowers at R_1_ (initiation of the flowering stage) were individually tagged using embroidery thread at appearance (Table 5). The thread of different colors was used for every date of flower initiation. In this way, each flower was followed. All the initiated and abscised flowers at each node were counted at each date, and then the total number of initiated and abscised flower per plant were calculated at the end of flowering. Then flower abscission (%) was calculated by using the following formulas^19^.

$$Flower\,abscission=\frac{Flower\,abscised}{Total\,flowers}\times 100$$

**Table 5:** Soybean physiological-stages and periods were recorded in 2017 and 2018.

Serial No.	Physiological stage	Growth period	DAS 2017*	DAS 2018*
1	Seed emergence	Germination	07	05
2	Fifth-trifoliate	Vegetative	35	33
3	Beginning-flowering	Pre-reproductive	51	54
4	Beginning-pod	Reproductive	64	66
5	Full-flowering	Reproductive	72	75
6	End-flowering	Reproductive	94	96
7	End-pod	Reproductive	101	103
8	Physiological-maturity	Reproductive	124	122
9	Full-maturity	Reproductive	136	134

*Plant samples were collected at 30, 50, 70 and 90 days after sowing (DAS) for all the measurements.

#### Yield and yield components

At full maturity, 40 consecutive soybean plants were harvested from the three plots per treatment (95% of pods achieved mature pod color). The pod number at each node of the main stem and the total number of pods plant^−1^ (PN) was determined. All the pods of the sampled plants were threshed manually, and then the seed number plant^−1^ (SN) was determined. The threshed seeds were weighed to determine the seed-yield plant^−1^ and changed into kg ha^−1^. Five lots of hundred soybean seeds from a bulk seed sample of every treatment were dried in an oven at 65 °C till constant weight reached, and then seed weight (SW) was determined using an electrical balance. Additionally, to evaluate the leaf removal effects on maize yield under MS_R_, we harvested the four m^2^ (twenty-four ears) from each treatment at maize maturity. All the harvested ears were dried under sunlight for 6 days; dried ears were manually threshed and weighed to determine the maize seed-yield of each treatment and then changed into kg ha^−1^.

#### Land equivalent ratio

The land equivalent ratio (LER) was calculated to determine the yield advantage of MS in response to different leaf removal treatments^60^:

$$LER=LERm+LERs=\frac{Yim}{Ysm}+\frac{Yis}{Yss}$$

Where LERs and LERm are the partial LER values for soybean and maize, respectively. Ysm and Yim are the seed-yield of maize (kg ha^−1^) under SM and MS, respectively. Yss and Yis are the seed-yield of soybean (kg ha^−1^) under SS and MS, respectively.

#### Statistical analysis

All the data recorded for every measured variable was analyzed using Statistix 8.1. Significance was determined via one-way analysis of variance. The least significance difference (LSD) test was employed to compare the means at 5% probability level. Microsoft Excel was used for the graphical presentation of data using standard error (±SE).

## Data Availability

The datasets generated during and/or analyzed during the current study are available from the corresponding author on reasonable request.
